# Impact of cucurbit crop management techniques on the foraging behavior of honeybees and hoverflies in Morogoro, Tanzania

**DOI:** 10.1186/s12862-024-02202-9

**Published:** 2024-01-17

**Authors:** Elvillah William Rweyemamu, Maulid Walad Mwatawala, George Muhamba Tryphone, Marc De Meyer, Sija Kabota, Patroba Masatu Bwire

**Affiliations:** 1 Department of Crop Science and Horticulture, Sokoine University of Agriculture (SUA), P.O. Box 3005, Chuo Kikuu, Morogoro, Tanzania; 2 Research, Consultancy and Publication Unit, National Sugar Institute (NSI), P.O. Box 97, Kidatu-, Morogoro, Tanzania; 3 Royal Museum for Central Africa, Invertebrates Section and JEMU, Leuvensesteenweg 13, B-3080 Tervuren, Belgium; 4 Tanzania Tobacco Board, P.O. Box 227, Morogoro, Tanzania

**Keywords:** GAMOUR-agroecology, Pollinators, Visitation frequency, Visitation rate

## Abstract

**Background:**

Poor agricultural practices have drastically threatened insect pollinators’ biodiversity. Little is known in Tanzania about how different agricultural practices affect pollinators’ foraging behavior. This study investigated the effects of the agroecological zone, season, cucurbit species and management practices on visitation frequency, visitation rate and time spent on cucurbit flowers by five pollinator species viz. *Apis mellifera*, *Eristalinus megacephalus*, *Mesembrius caffer*, *Paragus borbonicus* and *Toxomerus floralis.* The experiment was designed as a 5 × 3 × 3 × 2 × 2 factorial arrangement in a Randomized Complete Block Design (RCBD) with four replications. GAMOUR-Agroecology was tested against conventional practices and untreated control.

**Results:**

This study revealed significant effects of agroecological zone × season × cucurbit species × management practice on pollinators’ visitation frequency (*p* = 0.007) and time spent on flowers (*p* = 0.005). Also, agroecological zone × season × cucurbit species × pollinator species significantly (*p* < 0.0001) affected pollinators’ visitation frequency. Agroecological zones × season × cucurbit species × cucurbits management practices × pollinators significantly (*p* = 0.001) affected pollinators’ visitation rate. *Apis mellifera* was the most frequent visitor in *Cucurbita moschata* plots treated with GAMOUR- Agroecology in the plateau zone, also, visited higher number of *Cucumis sativus* plots under GAMOUR-Agroecology practices in the mountainous zone during the October–November season. Furthermore, it has been found that pollinators spent much in cucurbit flowers on plots with GAMOUR-Agroecology practices and control.

**Conclusions:**

Pollinators’ foraging behavior were enhanced by GAMOUR-Agroecology practices. Therefore, this study recommended that cucurbit growers should consider management practices that positively influence pollinator foraging activities for sustainable cucurbit production.

## Introduction

Insect pollinators are of economic importance in numerous cross-pollinated crops including cucurbits for sustainable food production [1]. They contribute to growth, improvement of fruit quality and setting of seeds [2]. This is because, for successful pollination, a female stigma has to be visited several times for the deposition of enough pollen [3]. Poor pollination service can result in a low fruit set, small, contorted fruit and in the absence of pollination, fruit abortion occurs [3–5]. Globally, about 85 to 90 % of cultivated flowering crops depend on insect pollinators for yield enhancement [6]. This has been estimated to contribute to about USD 200 billion annually to the global agricultural economy [7].

Cucurbits are among the fruit vegetables whose production is increasing worldwide due to an elevated market demand motivated by consumers’ health concerns [8]. However, the production of cucurbits is hampered by several factors including insufficient pollination services [9]. Pollination processes of most of cucurbit species require insect pollinators because, male flowers produce sticky and heavy pollen grains which are difficult to be carried by wind [2, 10]. The deficit of pollination services is linked to various issues, including poor agricultural practices that have reduced the number of insect pollinators and threatened their foraging behavior [11]. The global meta-analysis by the Intergovernmental Science-Policy Platform for Biodiversity and Ecosystem Services (IPBES) on the status and threats against pollinators and pollination has identified intensive agriculture as a major threat against pollinators [12]. Farm management directly affects the availability and quality of foraging and nesting resources for pollinators in the agricultural fields [13]. Since the 1960s, modern agriculture has rapidly intensified, and the dominant agriculture in many parts of the world uses large amounts of chemical fertilizers, pesticides and other technologies that direct or indirect affect insect pollinator biodiversity negatively [14–16].

Many studies have explored the effect of different agricultural management practices on insect pollinator biodiversity. For example, the investigation by Kovács-Hostyánszki et al. [17], Montoya et al. [18] and Karamaouna et al. [11] revealed that a reduction inorganic fertilizer and pesticides coupled with a more diverse edge vegetation have substantially affected pollinator communities with subsequent positive effects on agricultural productivity. According to Deguine et al. [19] GAMOUR-Agroecology practice in cucurbits is environmental agricultural approach which creates suitable habitats that provide food and shelter to pollinators and natural enemies of insect pests in agroecosystems. GAMOUR-Agroecology is an approach that was developed to manage fruit flies of cucurbits in the Reunion Island. The package aimed at improving soil health, the habitat of natural enemies, pollinators and increasing plant biodiversity in agroecosystems. Sanitation, trap plants, mass trapping and augmentation biological control were among the pillars of GAMOUR-Agroecology assessed during the implementation of the project [20].

In Tanzania, the emphasis on the agricultural sector is greatly given to soil fertility improvement, pest control and irrigation water availability [21]. On the other hand, the production of cucurbits rely heavily on conventional practices which pose negative impacts to both insect pollinators and consumers [22, 23]. Less attention has been paid to insect pollinator conservation during crop production. This was revealed from the survey conducted by Mkenda et al. [24] and Sawe et al. [25] in Northern Tanzania which documented that the majority of the local farmers are aware of neither the role of pollinating insects nor their conservation strategies and unable to distinguish beneficial insects from insect pests. Sawe et al. [21] pointed out the importance of pollinators in cucurbit production while, Lasway et al. [6] reported how agricultural intensification affects pollinator diversity. Still there was a need to investigate how different management practices affect the cucurbit-pollinator interaction, in order to come up with the best management practices for sustainable production. Therefore, this study assessed the effects of GAMOUR-Agroecology (new approach in the country which is environmentally friendly) and conventional practices (common method) in cucurbits production on the foraging behavior of pollinators. The foraging behavior of pollinators were assessed in terms of visitation frequency (number of visits on a single cucurbit flower by individual pollinator), visitation rate (number of cucurbit flowers visited by individual pollinator) and handling time (time spent on a flower by individual pollinators). We focused on five major insect pollinators in the region as revealed by Kabota [26]. These included: - *Apis mellifera* Linnaeus, *Eristalinus megacephalus* Rossi*, Mesembrius caffer* (Loew), *Paragus borbonicus* Macquart *and Toxomerus floralis* (Fabricius). We hypothesized that pollinators foraging behavior on cucurbit flowers are affected by crop species, altitude, season and their interaction. Since agroecology is a production method that encourages biodiversity and natural pest control, we further hypothesized GAMOUR-Agroecology practice positively impacts pollinator foraging behavior.

## Material and methods

### Description of the study area

The study was carried out in two agroecological zones of Morogoro region, Eastern-Central Tanzania, namely Plateau and Mountainous, from March to June 2021 (rainy season) and September to November 2021 (dry season). The area’s climatic conditions are characterized by a bimodal rainfall pattern, with short rains from October to December and long rains from March to May. Elevation and average annual rainfall in the mountainous zone ranges from 800 to 2000 m a.s.l and 1000 to 1200 mm respectively, whereas in the plateau zone ranges from 200 to 600 m a.s.l and 800 to 1000 mm respectively. During the period of study for the May–June season, the average temperature and relative humidity ranged, respectively, from 18 °C to 24 °C and 70 to 89% in the mountainous zone, and from 22 °C to 25 °C and 69 to 82% in the plateau zone. While during the October–November season, average temperature and relative humidity ranged, respectively, from 20 °C to 25 °C and 70 to 80% in the mountainous zone, and from 26 °C to 30 °C and 63 to 71% in the plateau zone. The experimental plots were established between 06°47′26″S 37°38′08″E and 06°51′01″S 37°39′17″E, in the plateau zone and between 06°52′32″S 37°40′16″E and 06°53′20″S 37°40′14″E, in the mountainous zone.

### Experimental layout and crop establishment

An experiment was designed as a 5 × 3 × 3 × 2 × 2 factorial in a Randomized Complete Block Design (RCBD) with four replications. Where five pollinating species (*A. mellifera, E. megacephalus, M. caffer*, *P. borbonicus and T. floralis*) were assessed under three cucurbit management practices (GAMOUR- Agroecology, Conventional and control) on three cucurbit species; cucumber (*Cucumis sativus* Linnaeus), watermelon (*Citrullus lanatus* (Thunb.) Matsum. & Nakai) and squash (*Cucurbita moschata* Duchesne), in the two agroecological zones of Morogoro (Plateau and Mountainous zone) for two cucurbit growing seasons (March–June and September – November 2021 (rainy and dry season, respectively)).

GAMOUR-Agroecology practices involved the use of border crop (maize) for attracting insect pollinators and trapping cucurbit fruit flies, mass trapping and killing of fruit flies using success bait GF120 (Spinosad 0.24 g/l) and Bio lure placed on border crop, use of organic mulches, use of augmentorium for field sanitation, and the use of organic fertilizers (farmyard manure). GF120 was sprayed on the border crop at the interval of seven (7) days from when the cucurbits started to flower (24th April, 2021 and 8th October, 2021, for rainy and dry, respectively) until 7 days before harvest. We mixed GF120 and water at a dilution ratio of 1 L GF120:7 L water. 0.016 L of a mixture was sprayed on the upper side of maize leaves on a 0.4 m^2^ spot and after every 10 m. Bio lure was also placed on the border crop and changed weekly until 7 days before harvesting. Conventional practices involved the use of insecticide AMADINE 40EC (Dimethoate 400 g/l, manufactured by Shandong Binnong Technology Co. Ltd), fungicide DACONIL® 720SC (Chlorothalonil 720 g/l, manufactured by Syngenta Crop Protection Ag-Switzerland) with the application rate of 2.5 L/Ha and 2.0 L/Ha, insecticide and fungicide, respectively and industrial fertilizers (N: P: K 15:9:20 and CAN 15:4:26, basal and top dressing fertilizers, respectively). A tank mix of fungicide and insecticide was sprayed 6 times per season at the interval of 14 days from when 75% of the crops had emerged (4th April, 2021 and 15th September, 2021 for rainy and dry season, respectively) up to 14 days before harvest. In a 20 L sprayer tank, 0.125 L and 0.05 L of Chlorothalonil and Dimethoate, respectively, were mixed in a 20 L of water and sprayed on the cucurbits. Plots without any management practices except irrigation during the dry season and weeding were used as controls. Watermelon “crimson sweet” variety, Cucumber “Ashley” variety, Squash “Waltham butternut” variety and Maize “Tumbili” variety used in this study were purchased from Agro-dealer in Morogoro town.

Sowing of maize (as a border crop in the GAMOUR fields) was done 30 days prior to the sowing of cucurbit seeds. The spacing of maize and cucurbits were 0.3 m × 0.3 m and 1.5 m × 1.0 m, respectively. One seed and two seeds per hole for maize and cucurbits respectively, were sown. The investigation started from 23rd March 2021 for the rainy season and 2nd September 2021 for the dry season on a 45 m × 45 m plot per treatment. Each experimental plot contained the three cucurbit species per specific management practice was located at least 100 m away from each other to avoid spray drift from the conventional plots.

### Assessment of honeybees’ and hoverflies’ foraging activities

The assessment of the foraging activities of the cucurbits flowers visitors began when the crop attained 10% flowering and continued until the end of the flowering period.

The investigation was conducted through visual observation in three phases between 0800 and 1700 hours, i.e. at 0800–0900, 1200–1300 and 1600–1700 hours on eight spots of 4 m^2^ each which were established after every 5 m within the experimental plot per cucurbit species per management practice. The observations were conducted weekly following the procedures adopted from Meerabai [27] and Yogapriya et al. [28].

### Data collection

We assessed both the abundance of pollinators and their visitation frequency. To measure pollinator abundance, we counted the total number of pollinator species visiting the cucurbit flowers. Visitation frequency was determined by closely observing a single flower for up to 1 minute and recording the number of visits made by each pollinating species. On each cucurbit management practice, a total of 72 flowers were observed per day in which 24 flowers were selected from each cucurbit species. An average number of visits on a single cucurbit flower per cucurbit species per management practices by individual pollinating species was calculated by the formula described by Zameer et al. [7]:

1 $$VF=\frac{\textrm{TNV}}{\textrm{TFO}}$$

Where: VF is visitation frequency, TNV is total number of visits and TFO is the total number of flowers observed.

Visitation rate was determined by tracking an individual pollinating species for a maximum of 1 minute from the time it arrived on the first flower within a 4 m^2^ spot and the following information was recorded: the total number of flowers visited, time (seconds) spent on each visited flower and time spent in flight between consecutive flowers. A total of nine observations were performed for each pollinating species per cucurbit management practice per day in which each pollinating species was observed three times per cucurbit species. An average number of flowers visited per pollinating species was determined following the formula described by Meerabai [27]:

2 $$VR=\frac{\textrm{TNFV}}{\textrm{TSF}+\textrm{TFBCF}}$$

Where: VR is the visitation rate, TNFV is the total number of flowers visited, TSF is the total time spent on flowers and TFBCF is the time in flight between consecutive flowers.

The average time spent on each visited flower (handling time) was processed as per the formulas described by Meerabai [27].

3 $$HT=\frac{\textrm{TSF}}{\textrm{TNFV}}$$

Where: HT is handling time, TSF is the total time spent on flowers and TNFV is the total number of flowers visited.

### Statistical analysis

The processed response variables were subjected to normality test using Shapiro-Wilk test. Since they were not normally distributed, then, the analysis of variance (ANOVA) was performed using Generalized Linear Mixed Models (GLMMs) procedures to check the effect of agroecological zones, seasons, cucurbit species and cucurbits management practices on honeybees and hoverflies foraging behavior at a 5% level of confidence. This is because, GLMMs incorporate non normal data that involve both fixed and random effects without transformation [29]. Fixed factors included agroecological zones, seasons, cucurbit species, cucurbits management practices and their interactions while, sampling week was used as a random factor. We performed model selection using Akaike’s information criterion. To validate the significance of the factors post hoc test was performed, and means were compared using Tukey’s Honest Significant Difference at 5%. All statistical analyses were carried out using R software version 4.1.0 [30].

## Results

### Abundance of honeybees and hoverflies on cucurbit flowers across the three cucurbits management practices

A total of 43,510 visit counts of honeybees and hoverflies were recorded on cucurbit flowers for the whole study period in all the plots of each management practice (GAMOUR-Agroecology, conventional and control. The number of visits by individual flowers visitors was in the proportion of 60.3% *A. mellifera*, 25.14% *T. floralis*, 7.56% *P. borbonicus*, 6.0% *E. megacephalus* and 1.0% *M. caffer*.

### Effects of agroecological zones, seasons, cucurbit species and cucurbits management practices on the visitation frequency

**Fig. 1:**
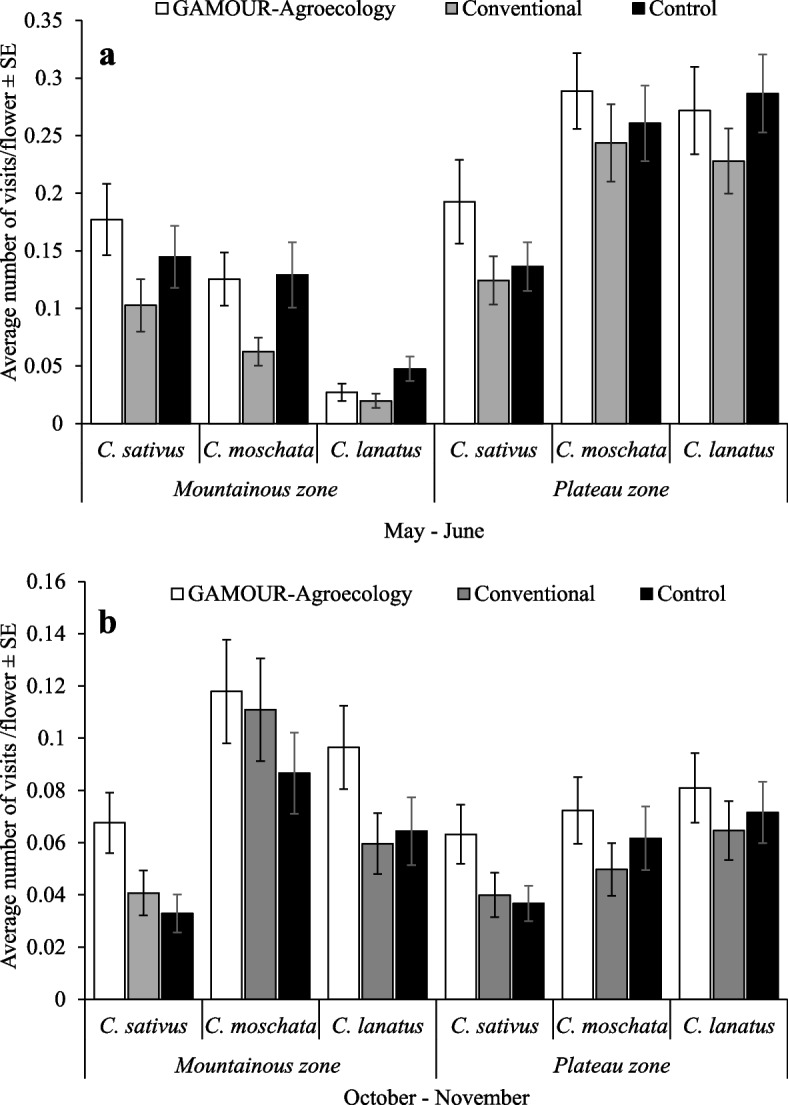
**a** and **b** Visitation frequency of pollinators on cucurbit flowers as affected by agroecological zones, cucurbit species and management practices

Our results showed non-significant effects of the interaction of agroecological zone, season, cucurbit species, management practice and pollinator species on the visitation frequency of the pollinators. However, the four-way interactions: agroecological zone × season × cucurbit species × management practice had significant (F_4, 5033_ = 3.56, *p* = 0.007) effects on visitation frequency of pollinators on cucurbit flowers (Fig. 1a and b). Further examination showed the variations in visitation frequency were significant within both seasons. We found that visitation frequencies in *C. moschata* and *C. lanatus* plots in the plateau zone, during the May – June season were not significantly different among management practices, but were significantly higher than the visitation frequencies in all practices and cucurbits in the mountainous during the May – June season and in both zones during October–November season. Generally, the highest visitation frequency was recorded in *C. moschata* plots treated with GAMOUR- Agroecology in the plateau zone, while the least was recorded in *C. lanatus* plots treated with conventional practices in the mountainous zone during May–June season.

**Fig. 2:**
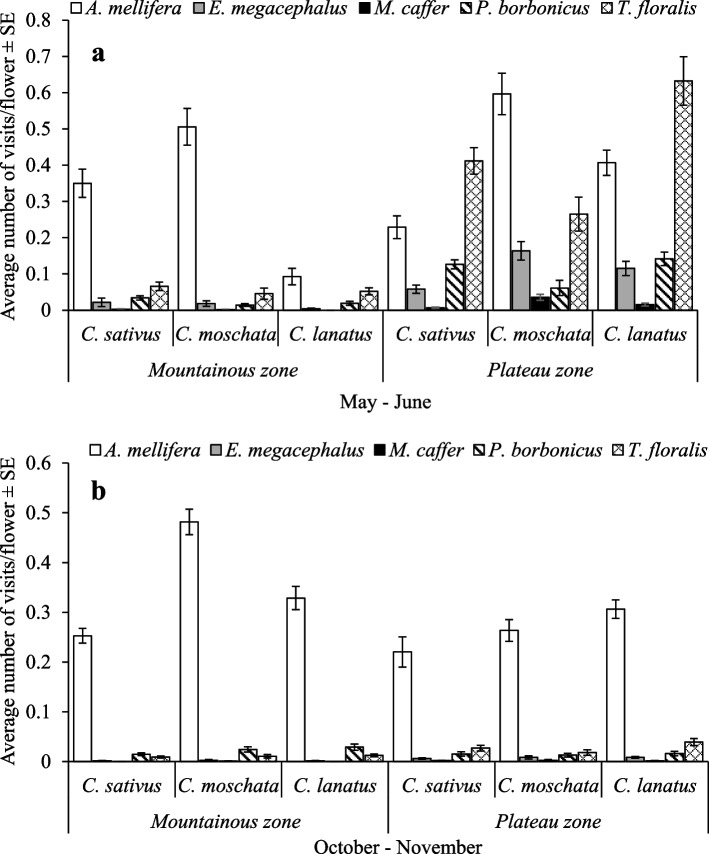
**a** and **b** Visitation frequency of pollinators on cucurbit flowers as affected by agroecological zones and cucurbit species

The effects of agroecological zone × season × cucurbit species × pollinator species were also significant (F_8, 5033_ = 7.42, *p* < 0.0001) (Fig. 2a and b). *Apis mellifera* was significantly the most frequent visitor during both seasons and agroecological zones (. A notable exception was the visitation frequency of *T. floralis* to *C. lanatus* plots, in the plateau zone during the May – June season (Fig. 2a).

### Effects of agroecological zones, seasons, cucurbit species and cucurbits management practices on the visitation rate

**Fig. 3:**
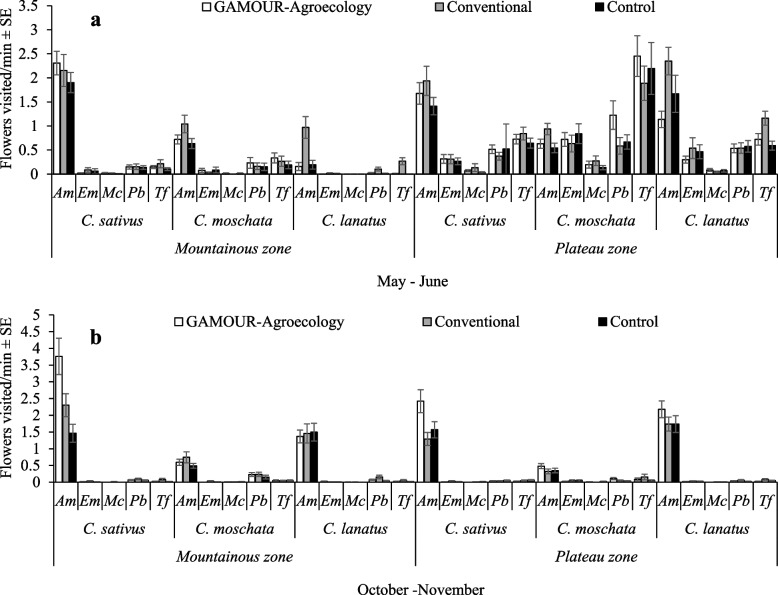
**a** and **b** Visitation rate of pollinators on cucurbit flowers as affected by agroecological zones, cucurbit species and management practices. Key: *Am* is *A. mellifera*, *Em* is *E. megacephalus*, *Mc* is *M. caffer, Pb* is *P. bobornicus* and *Tf* is *T. floralis*

There were significant effects of the five-way interaction of agroecological zone × season × cucurbit species × cucurbits management practice × pollinator (F_16, 5033_ = 2.55, *p* = 0.001) on the visitation rate of pollinators on cucurbit flowers. The variations were significant within both seasons. Visitation rates by *A. mellifera* in *C. sativus* plots were similar among management practices but were significantly higher than all other combinations in the mountainous zone). A notable exception was the visitation rate of *T. floralis* to *C. moschata* plots, in the plateau zone during the May – June season (Fig. 3a). A similar situation was observed in the October – November season, rates by *A. mellifera* were significantly higher regardless of zone and cucurbit species. Variations among practices, in that case, were not significantly different except in *C. sativus*. Highest visitation rates were revealed by *A. mellifera* in *C. sativus* plots under GAMOUR-Agroecology practices in the mountainous zone during the October–November season (Fig. 3b). In contrast, visitation rates were the lowest for *E. megacephalus* and *M. caffer* in *C. lanatus*, under all practices in the mountainous zone during both seasons (Fig. 3a and b)

### Effect of agroecological zones, seasons, cucurbit species and cucurbits management practices on thehandling time

**Fig. 4:**
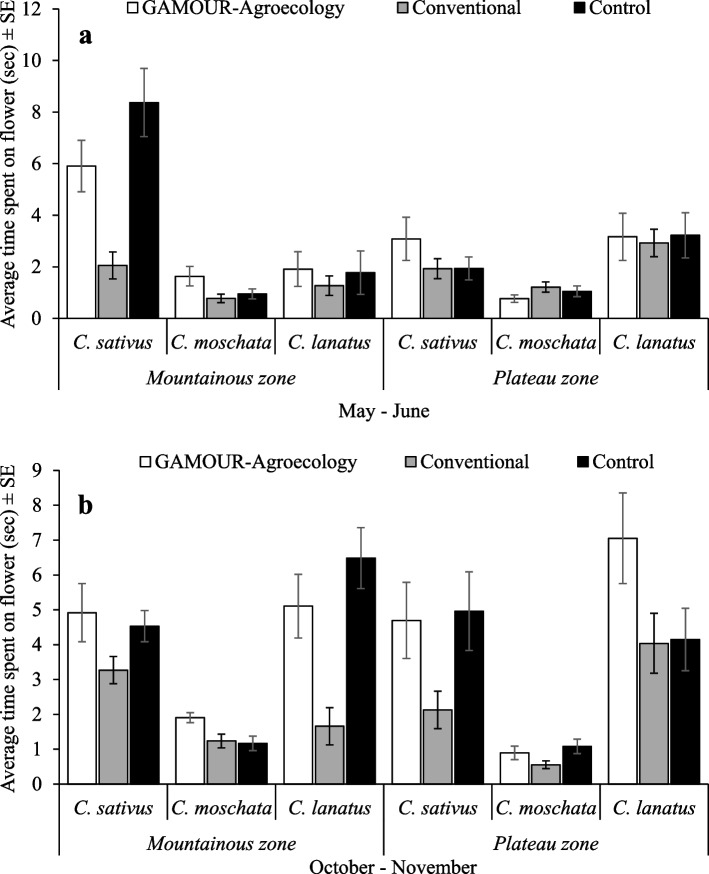
**a** and **b** Time spent on flowers by pollinators as affected by agroecological zones, cucurbit species and management practices

The four-way interactions of agroecological zone × season × cucurbit species × management practice significantly (F_4, 5040_ = 3.78, *p* = 0.005) affected the time spent by pollinators on cucurbit flowers. The differences in time spent by pollinators were significant among factors when analyzed by season. Time spent by pollinators in *C. sativus* under control in the mountainous zone, during the May–June season was significantly the longest (Fig. 4a). Furthermore, time spent by pollinators in crops under GAMOUR - Agroecology and control practices was statistically similar, but significantly higher than in crops under conventional practices during the October – November cropping season (Fig. 4b). Exceptions were time spent in *C. lanatus* in plateau zone and *C. moschata* in mountainous zone.

In general, pollinators spent more time in *C. sativus* under control in the mountainous zone, and the least time in *C. moschata* under conventional and GAMOUR-Agroecology practices, in both zones During the May–June season (Fig. 4a). Furthermore, during the October–November season, pollinators spent more time in *C. lanatus* under GAMOUR-Agroecology practices in the plateau zone and the least time in *C. moschata* under conventional practices in the plateau zone (Fig. 4b).

**Fig. 5:**
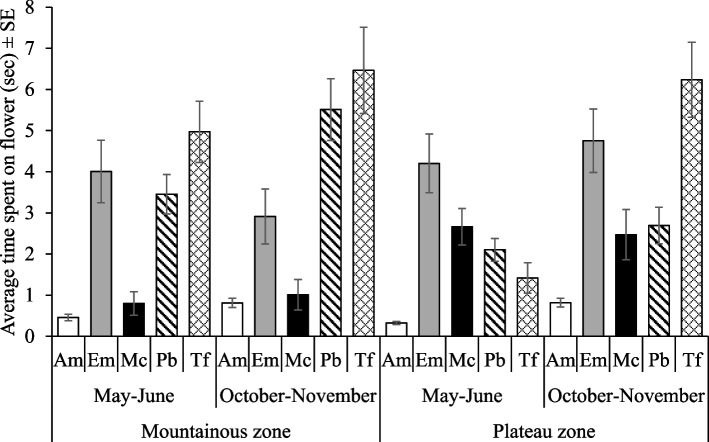
Time spent on flowers by pollinators as affected by agroecological zones and season. Key: *Am* is *A. mellifera*, *Em* is *E. megacephalus*, *Mc* is *M. caffer, Pb* is *P. bobornicus* and *Tf* is *T. floralis*

**Fig. 6:**
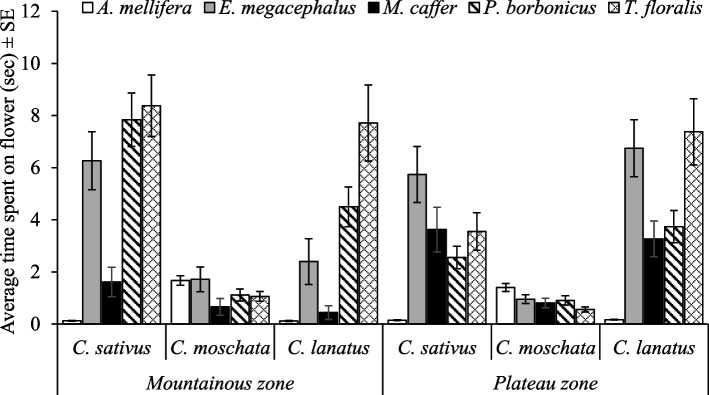
Time spent on flowers by pollinators as affected by agroecological zones and cucurbit species

**Fig. 7:**
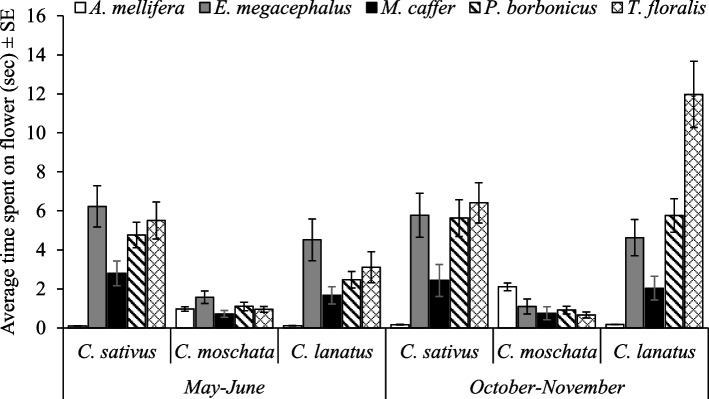
Time spent on flowers by pollinators as affected by season and cucurbit species

**Fig. 8:**
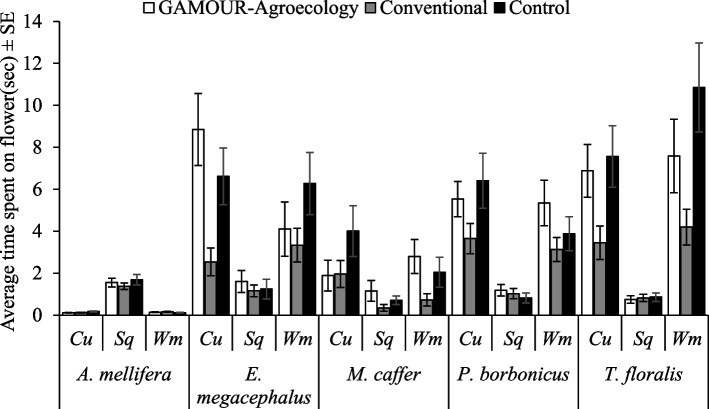
Time spent on flowers by pollinators as affected by cucurbit species and management practices. Key: Cu is *C. sativus*, Sq is *C. moschata* and Wm is *C. lanatus*

Furthermore, three-way interactions involving pollinator species as a factor, we found that;. 1) Time spent by a pollinator species was significantly dependent on agroecological zone × season (F_4, 5040_ = 2.93, *p* = 0.02) as well as agroecological zone × cucurbit species (F_8, 5040_ = 3.81, *p* < 0.0001). *T. floralis* spent more time in *C. sativus* and *C. lanatus* in the mountainous zone during the season October–November and the least time by *A. mellifera* in *C. sativus* in the plateau zone during the season of May – June (Figs. 5 and 6). 2) Season × cucurbit species also determined the time spent by a pollinator species on cucurbit flowers (F_8, 5040_ = 5.26, *p* < 0.0001). The time spent by *T. floralis* in *C. lanatus* was significantly higher than the time spent by the rest with the lowest time spent by *A. mellifera* in *C. sativus* during the season of May – June (Fig. 7). 3) The time spent by a pollinator species significantly (F_16, 5040_ = 2.06, *p* = 0.007) varied with cucurbit species × management practices (Fig. 8).

## Discussion

The present study found that honeybees were abundantly visiting cucurbits than hoverflies in each experimental plot. This is because, honeybees are major pollinators of cross-pollinated crops including cucurbits and it has been discovered to contribute 80% of insect pollination [31, 32]. Pollinating insects improve the quality and quantity of cucurbits [33]. However, this study mainly focused on the determinant factors of cucurbits quality and quantity which were visitation frequency, visitation rate and time spent on cucurbit flowers. The contribution of pollinators to pollination on cucurbits was beyond the scope of this study.

Visitation frequency of honeybees and hoverflies on cucurbit flowers was significantly affected by interactions of the agroecological zone, season, cucurbit species and management practice. Likewise, the interaction of agroecological zone, season, cucurbit species and pollinator species significantly affected their visitation frequency on cucurbit flowers. All cucurbit flowers per management practice were more frequently visited by pollinators during May – June season in the plateau zone compared to the rest. In the mountainous zone and during October –November, pollinator abundance had low number because of the presence of few flowers compared to the plateau zone [34], this translated to fewer visits. Pollinator abundance and visitation frequency to flowers are influenced by weather conditions [35–37] and the presence of floral resources [38–40]. These findings correlate with the findings in the studies by Doyle et al. [41], Mertens et al. [42] and Pi et al. [43] who reported the presence of a low number of pollinators at a higher elevation and during the dry season. Furthermore, the visitation frequency was higher in cucurbits under GAMOUR-Agroecology practices and control. This is because, the presence of flowering border crops and lack of use of synthetic inputs provided additional floral resources and created favorable conditions for pollinators hence contributing to their visitation frequency [44–48].

The study findings also revealed significant effects of the interaction of agroecological zones, season, cucurbit species, cucurbits management practice and pollinator species on visitation rates. The visitation rate of *A. mellifera* was significantly higher in *C. sativus* plots under GAMOUR-Agroecology practices in the mountainous zone during the October–November season compared to the rest. The high visitation rate of *A. mellifera* is attributable to its feeding mechanism involving the engagement of several bee workers to exploit the nutritional resources of the colony [49]. Also, the higher visitation rate of *A. mellifera* in plots under GAMOUR-Agroecology occurred because of the use of low synthetic inputs and additional flower strips at the borders creating a pollinator-friendly habitat thus, stimulating their foraging activities [50–52]. Pesticide application in the fields when coming into contact with insect pollinators reduces their foraging performance including flight time and visitation rate [53, 54]. Likewise, the pollen/nectar on flowers treated with pesticides have higher neonicotinoids residuals which consequently have negative effects on the central nervous system of pollinators [47, 52, 55].

From this study, we further found that the interaction of agroecological zone, season, cucurbit species and management affected the time spent on cucurbit flowers by honeybees and hoverflies. Pollinators spent more time on cucurbit flowers on the control plots and the plots under GAMOUR-Agroecology in which *A. mellifera* stayed longer on *C. moschata* flowers and hoverflies on *C. lanatus* and *C. sativus* flowers. The shorter time spent by pollinators on cucurbit flowers on the plots were synthetic inputs were applied could be related to the deterrent of pollinators to the residual effect of pesticides. This finding is consistent with findings in other studies which reported that systemic pesticide application to crops translocate throughout the plant tissues and accumulates in plant nectar and pollen, thus once taken by pollinators it brings negative effects on their foraging behavior [56–59].

Time spent on a crop by a pollinator species was significantly dependent on season and cucurbit species as well as season and agroecological zone. Furthermore, interactions between cucurbit species and agroecological zone as well as cucurbit species and management practices affected pollinator species’ time spent on crops. *A. mellifera* spent more time on *C. moschata* flowers due to the availability of more nectars compared to in *C. lanatus* and *C. sativus* flowers because honey bees prefer more nectar to pollen [3, 60]. Hoverflies spent more time on *C. lanatus* and *C. sativus* flowers than on squash flowers due to the easy accessibility of pollens [61, 62].

## Conclusions

In conclusion, we found that cucurbit management practices affected the number of flowers visited by pollinators and the time spent on the individual flowers but had a slight effect on the number of visits per cucurbit flower. GAMOUR - Agroecological practices enhanced the foraging activities of pollinators. Agroecological zones, and seasons also significantly affected the foraging behaviour of pollinators. The number of visits per flower and the number of flowers visited was higher in the plateau zone during the May–June season. Therefore, cucurbit growers should consider management practices that might positively influence pollinator foraging activities for sustainable cucurbit production and grow cucurbits in the plateau zone during the season of May–June when pollinators are abundantly available.

## Data Availability

All data supporting the findings of this study are available at https://osf.io/kt74b/?view_only=5584a49305304a95b5b3df19698f263d.
